# Transcriptomics and Metabolomics Explain the Crisping Mechanisms of Broad Bean-Based Crisping Diets on Nile Tilapia (*Orechromis niloticus*)

**DOI:** 10.3390/metabo14110616

**Published:** 2024-11-12

**Authors:** Xiaogang He, Haoming Shu, Tian Xu, Minhui Yu, Honglin Li, Yanru Hu, Jiajun Mo, Chunxiang Ai

**Affiliations:** 1 College of Ocean & Earth Sciences, Xiamen University, Fujian 361005, China; 2 Anyou Biotechnology Group Co., Ltd., Jiangshu 215400, China; 3 Marine and Fishery Institute of Xiamen, Fujian 361000, China; 4 State Key Laboratory of Mariculture Breeding, Xiamen 361102, China

**Keywords:** broad bean, crisping diet, Nile tilapia, metabolism reprogramming, glucose metabolism

## Abstract

**Background/Objectives:** To investigate the crisping mechanism of broad bean-based crisping diets on Nile Tilapia. **Methods:** Four crisping diets were designed to feed 360 fish for 90 days, and multiomics analyses were employed. **Results:** Our results indicated that the designed crisping diets for Nile tilapia can effectively make tilapia muscle crispy. The ingestion of broad bean-based diets induced metabolic reprogramming dominated by glycolytic metabolism inhibition in fish, and metabolic reprogramming is the initiator of muscle structural remodeling. Among these, glucose is the main DAMP to be recognized by cellular PRRs, activating further immune response and oxidative stress and finally resulting in muscle change. **Conclusions:** Based on our results of multiomics, *pck2*, and *ldh* played main roles in crisping molecular mechanisms in driving the initial metabolic reprogram. Moreover, the addition of the crisping package further activated the ErbB signaling pathway and downstream MAPK signaling pathway to strengthen immune response, promoting muscle fiber development and growth. Our study delved into the effects of crisping formula diet on the liver of Nile tilapia at the molecular level, providing theoretical guidance for the nutritional regulation of crispy Nile tilapia.

## 1. Introduction

Tilapia (*Oreochromis* sp.), also known as African crucian carp, is native to Africa and the Middle East [1]. It is a tropical fish that inhabits the middle and lower layers of water and has an omnivorous diet mainly composed of aquatic plants [2]. Cultivated tilapias are mainly Nile tilapias, which have been widely introduced (over 100 countries and regions) and have the highest aquaculture yield, accounting for approximately 70% of the world’s total production [3]. Nile tilapia has a wide range of food habits, strong adaptability to the environment, good disease resistance, strong reproductive ability, high population yield, delicious meat taste, and high demand in the international market [4]. However, due to the lower market price of tilapia, it is crucial to find a way to improve the quality of its production and raise its value [5]. One possible solution could be to improve its flesh quality, such as with crispy carp.

The broad bean, *Vicia faba* Linn (Fabales, Fabaceae), is an annual herbaceous plant. The main nutritional component of broad beans is starch, which accounts for approximately 50% to 60% of dry matter, followed by protein, which accounts for approximately 25% to 35% of dry matter [6]. Due to their relatively high nutritional values, broad beans can partially replace wheat flour and fish meal as feed raw materials. At present, some fish farming uses broad beans to change the muscle structure of farmed fish and improve their muscle hardness, chewiness, and collagen content, thereby improving the muscle quality of farmed fish and increasing their economic value [7,8,9]. So far, broad beans have become a special feed material for meat crispiness. However, although the proportion of essential amino acids and the total amino acids are equivalent to that of soybeans, methionine is the limiting amino acid. Therefore, when used as feed material, additional methionine should be supplemented [10].

In addition to the deficiency of methionine in leguminous plants, broad beans also present high molecular weight condensed tannins, low molecular weight polyphenols, trypsin inhibitors, phytic acid, lectins, and other antinutritional factors [11]. As a result, the growth performance of fish is usually negatively impacted when given broad bean-containing feed, which can easily trigger inflammatory reactions in fish, damaging their liver and intestines, reducing their antioxidant capacity, and increasing the risk of death. These disadvantages restrict the application of broad beans in cultivating crispy fish. Pre-processing methods, such as peeling, soaking, heat treatment, germination, and enzyme addition, are commonly applied to reduce or inactivate the antinutritional factors of broad beans [12,13,14]. Developing efficient and safe formula feed for crispy fish will help promote the healthy development of the crispy fish industry.

Research has found that feeding tilapia with broad beans can significantly improve its meat quality [15,16]. Developing a crisping formula diet that can achieve muscle crispness is crucial for the development of tilapia aquaculture. The currently developed crisping formula diet leads to some side effects; it could significantly reduce the growth performance of tilapia while also causing damage to the liver tissue structure and reducing its antioxidant capacity [17]. The liver, as the metabolic center of the body, plays an important role in maintaining metabolic homeostasis [18]. However, the metabolic response of the liver and the adaptive mechanism to broad beans are still unclear for tilapia, and that was the basis for improving and perfecting the application of broad beans.

Two broad bean-based crisping diets were designed to determine the effects of broad bean supplementation on Nile Tilapia. In addition, another two experimental diets with a crisping functional package were applied to eliminate the negative impacts of broad beans as a feed supplement. Our previous research has indicated that all these four designed diets reached the crisping effect while having no adverse effect on the growth performance and that the supplement of the crisping functional package can further improve the crisping effect (especially the G5 diet) [19]. In addition, the crisping functional package also helped to improve the intestinal tissue and microbiota structure. Based on these preliminary results, multiomics techniques (transcriptome and metabolome) were employed to further explore the inherent crisping mechanism. This study will delve into the effects of crisping formulated diet on the liver of Nile tilapia at the molecular level, providing theoretical guidance for the nutritional regulation of crispy Nile tilapia.

## 2. Materials and Methods

### 2.1. Experimental Diet

Six experimental diets were used in the present study; the formulation of diets (G1–G6) was according to He et al. [19]. A basic diet without broad bean and crisping functional feed additive mixture (CFFAM; formula is based on the research by He et al. [19]) was set as the control, while a commercial diet was used as the positive control (G6). Experimental diets G2 and G3 contained 40% and 50% of additional broad bean meal as the plant-based protein source, respectively. Then, the contents of lysine and methionine (the first and second limiting amino acids of tilapia) were balanced. In addition, a crisping functional feed additive mixture (CFFAM) was added at 0.5% dry weight into the G2 and G3 diets, respectively, as treatments G4 and G5. Raw materials were crushed and sieved through an 80 mesh sieve before being mixed. Then, about 40% of distilled water (*w*/*w*) was added to the mix to produce puffed diets at a diameter of 3.0 mm. Diets were dried at 60 °C for 24 h and put into the −20 °C refrigerator for storage.

### 2.2. Experimental Fish and Feeding Management

Nile tilapias were provided by the Guokeng Nile tilapia farm (Zhangzhou, Fujian, China). All fish were from the same breeding batch. After being transported to the laboratory, fish were fed the G1 diet for 7 days for acclimation.

After acclimation, a total of 360 fish (body weights of 617.32 ± 1.64 g) were randomly allocated to 24 barrels (1 m diameter, 1.3 m water depth). Four replicates were set up for each treatment (six treatments in total, five fish per replicate). The feeding experiment lasted 90 days, during which G1–G6 diets were served. During the feeding experiment, fish were fed twice daily (at 8:00 and 16:00) at 1.0–1.5% of their body weight. The water temperature was kept at 25 to 28 °C, pH was between 6 and 7.5, and dissolved oxygen was maintained above 6.0 mg/L throughout the feeding period.

### 2.3. Sampling and Analyzing

#### Sampling

At the end of the feeding experiment, four fish per barrel were randomly selected, and their liver was dissected and collected in 1.5 mL sterile enzyme-free centrifuge tubes, frozen in liquid nitrogen, and then quickly transferred to a −80 °C refrigerator. Two liver samples per barrel (six per group) were used for transcriptome analyses, and the other two for metabolome analyses.

### 2.4. Liver Transcriptome Analysis

#### 2.4.1. RNA Extraction and Sequencing

The total RNA of liver samples from G1 to G6 diet groups was extracted using the Trizol method, and quality control was performed on the total RNA. After passing the quality control, magnetic beads with oligo (dT) were used to enrich the mRNA in the sample. After that, a fragmentation buffer was added to break the mRNA into short fragments. Using random primers, mRNA was used as a template for reverse transcription to synthesize one-stranded cDNA, followed by the synthesis of two-stranded cDNA. We purified the double-stranded cDNA, performed end repair, and added A and adapter on the double-stranded cDNA. AMPure XP beans were used for double-stranded cDNA fragment size selection. Finally, a cDNA library was constructed through PCR amplification. After the library passed the quality test, Illumina high-throughput sequencing was performed.

The adapter sequence and low-quality reads of the original sample data were filtered, followed by a quality evaluation, transcript assembly, and unigene functional annotation according to the Kyoto Encyclopedia of the Genes and Genomes database (KEGG, accessed on 15 September 2022, http://www.genome.jp/kegg/).

#### 2.4.2. Differential Expression Analysis and Function Enrichment

By comparing the transcriptome data of samples from different groups, differential genes were screened. The hypothesis testing probability (*p* value) was calculated through statistical models. Finally, multiple testing was performed with the Benjamin & Hochberg method (BH) to obtain the FDR value (False discovery rate). Using the Reads per kilobase million (RPKM value), the multiple differences of the gene between samples were calculated. KEGG enrichment analysis was conducted on the genes to identify the metabolic pathways in which they are primarily involved.

### 2.5. Liver Metabolome Analysis

#### 2.5.1. Metabolite Extraction and LC-MS Analysis

The liver metabolites of tilapia were extracted using an extraction solution with methanol:acetonitrile (1:1). We shook and mixed samples after adding the extraction solution and sonicated them for 10 min. Then, they were kept at −20 °C for 1 h; after that, they were centrifuged at 13,000 rpm (4 °C, 15 min), and we vacuum-dried the supernatant. The extraction solution was added again, and the same operation process as above was performed again.

Final extracts were collected for LC-MS (liquid chromatography-tandem mass spectrometry) analysis to detect liver metabolites. LC was performed using the Acquity UPLC system (Waters, Milford, MA, USA) with an Acquity UPLC BEH Amide column (1.8 µm, 2.1 × 100 mm, Waters, Milford, MA, USA). The injection volume was 2 μL, the injector temperature was 4 °C, and the flow rate was 300 μL/min. The mobile phase was 100% H_2_O + HCOOH (A) and 100% acetonitrile (B) with gradient elution as follows: 0–12 min for 95% A and 5% B, 12–13.5 min for 5% A and 95% B, and 13.6–16 min for 95% A and 5% B; MS was performed with a Q Active mass spectrometer (Thermo Fisher Scientific, Carlsbad, CA, USA). Under the positive and negative ion modes, the heater temperature, sheath gas flow rate, auxiliary gas flow rate, exhaust gas flow rate, and capillary temperature were 300 °C, 45 arb, 15 arb, 1 arb, and 350 °C, respectively. Spray potentials of the positive and negative ion modes were 3.00 kV and 3.20 kV, respectively. The S-Lens RF levels were 30% and 60%, respectively. Full scanning was carried out with 70,000 (Mass spectrometry, MS) and 17,500 (MS/MS) resolutions. Then, high-energy collision dissociation (HCD) was used for secondary fragmentation.

#### 2.5.2. Data Preprocessing and Differential Metabolites Analysis

ProteoWizard software (v3.0.8789) was employed to convert data into a mzXML format, and the XCMS package was used for retention time correction, peak recognition, peak extraction, peak integration, peak filtering, peak alignment, etc. (cutoff was set to 0.6, minifrac was set to 0.5). We used a self-written R package and a self-built secondary mass spectrometry database (Ovison Gene Technology Co., Ltd., Beijing, China) for substance identification of peaks. According to OPLS-DA, when the first principal component of variable importance in projection (VIP) ≥ 1, and the *p* value of the Student’s *t*-test ≤ 0.05, the differential metabolites in different groups were screened. Based on the KEGG database, metabolic pathways were enriched for differential metabolites. Finally, the pheatmap package (version 1.0.10) of the R software (v3.3.1) and Spearman correlation analysis methods were used to analyze the correlation between the main differential functional genes and the main differential metabolites.

## 3. Results

### 3.1. Differential Genes Expression Analysis and KEGG Function Enrichment Based on Liver Transcriptome

**Figure 1:**
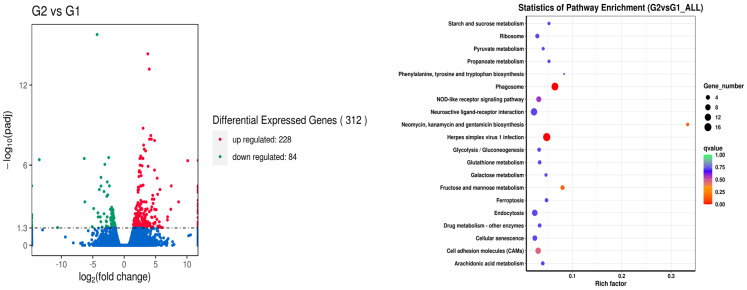
Analysis of liver gene difference between the G2 and G1 groups.

**Figure 2:**
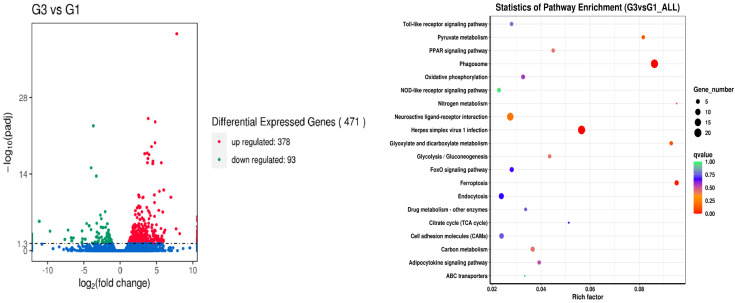
Analysis of liver gene difference between the G3 and G1 groups.

**Figure 3:**
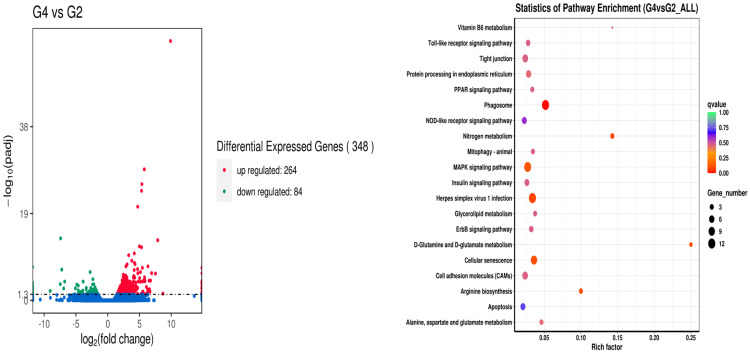
Analysis of liver gene differences between the G4 and G2 groups.

**Figure 4:**
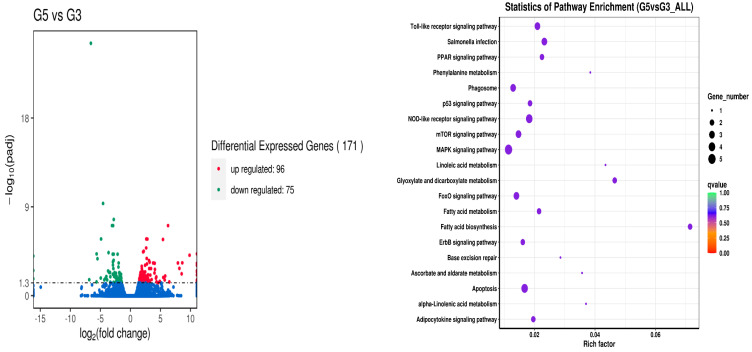
Analysis of liver gene differences between the G5 and the G3 groups.

**Figure 5:**
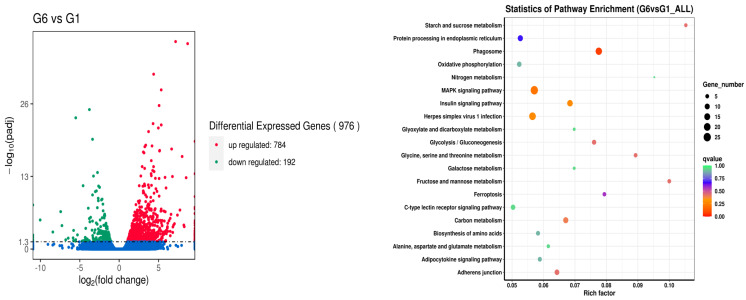
Analysis of liver gene difference between the G6 and G1 groups.

Results of the differential expression analysis of genes are presented in Figure 1, Figure 2, Figure 3, Figure 4 and Figure 5. When comparing the broad beans-free control (G1) and the low broad beans-containing treatment (G2, 40% of broad beans), 228 upregulated genes and 84 downregulated genes were identified. Among them, differences mainly enriched in the pathways of herpes simplex virus 1 infection, neomycin, kanamycin, gentamicin biosynthetic, fructose and mannose metabolism, and phagosome (Figure 1), with the most differential genes being *me1* (malate dehydrogenase) and *ldh* (L-lactate dehydrogenase). While comparing the control (G1) and the high broad beans containing treatment (G3, 50% of broad beans), more genes were identified as differential expression; the number of upregulated and downregulated genes were 378 and 93, respectively. In this set of comparisons, the gene differential expressions mainly concentrated in the pathways of nitrogen, carbon, pyruvate metabolism, neuroactive ligand-receptor interaction, ferroptosis, phagosomes, herpes simplex virus 1 infection (Figure 2), and the differential genes included *pck2*, *me1*, and *acss1*.

On the other hand, the results of the differential expression of genes among fish fed with 40% broad bean feed with or without crisping functional packages (i.e., treatments G2 and G4) revealed that 264 genes were upregulated, and 84 genes were downregulated. The metabolic pathways that had a significant impact were mainly the phagosome, Toll-like receptor signaling pathway, tight junction, protein processing in the endoplasmic reticulum, PPAR signaling pathway, mitophagy-animal, insulin signaling pathway, herpes simplex virus 1 infection, D-glutamine and D-glutamate metabolism, glycerolipid metabolism, ErbB signaling pathway, cellular senescence, cell adhesion molecules (CAMs), nitrogen metabolism, alanine, aspartate and glutamate metabolism, and MAPK signaling pathway (Figure 3). Compared with the G3, where fish were fed with 50% broad bean feed, the treatment with additional crisping package supply (G5) had 96 genes upregulated, and 75 genes downregulated, mainly presented in the fatty acid biosynthesis and metabolism, Toll-like receptor signaling pathway, PPAR signaling pathway, phagosome, NOD-like receptor signaling pathway, mTOR signaling pathway, glyoxylate, and dicarboxylate metabolism, FoxO signaling pathway, ErbB signaling pathway, MAPK signaling pathway, apoptosis and adipocytokine signaling pathway (Figure 4).

Finally, when comparing the broad bean-free control (G1) and the commercial crisping feed (G6, positive control), 784 upregulated genes and 192 downregulated genes were found in the latter. They were mainly presented in the phagosome, MAPK signaling pathway, insulin signaling pathway, herpes simplex virus 1 infection, starch and sucrose metabolism, fructose and mannose metabolism, glycine, serine, and threonine metabolism, carbon metabolism, adherens junction and glycolysis/gluconeogenesis (Figure 5).

### 3.2. Liver Metabolome

#### 3.2.1. Differential Metabolites Analysis

**Figure 6:**
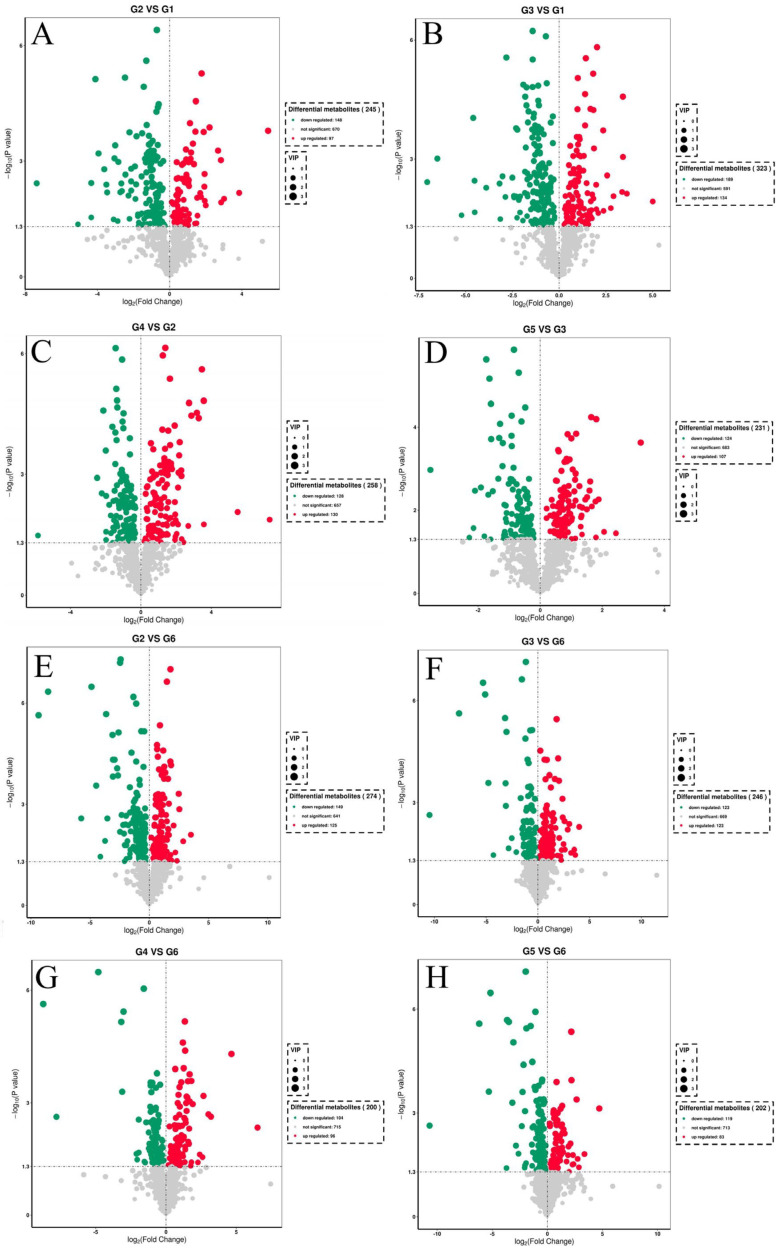
Volcanic maps of different metabolites in the liver of fish from different comparison groups. (**A**,**B**) volcanic map of differential metabolites in the liver between G2 or G3 and G1 groups; (**C**) volcanic map of differential metabolites between G2 and G4 groups; (**D**) volcanic map of differential metabolites between G3 and G5 groups; (**E**–**H**) volcanic map of differential metabolites between G2, G3, G4 or G5 and G6 groups.

The results of the differential metabolites in the liver metabolism of tilapia fed with different diets are shown in Figure 6. Compared with the G1 group, there were 97 upregulated metabolites and 148 downregulated metabolites in the G2 group. Compared with the G1 group, there were 134 upregulated metabolites and 189 downregulated metabolites in the G3 group. Compared with the G2 group, there were 130 upregulated metabolites and 128 downregulated metabolites in the G4 group. Compared with the G3 group, there were 107 upregulated metabolites and 124 downregulated metabolites in the G5 group. Compared with the G6 group, the G2 group had 125 upregulated metabolites and 149 downregulated metabolites. Compared with the G6 group, there were 123 upregulated metabolites and 123 downregulated metabolites in the G3 group. Compared with the G6 group, there were 96 upregulated metabolites and 104 downregulated metabolites in the G4 group. Compared with the G6 group, there were 83 upregulated metabolites and 119 downregulated metabolites in the G5 group.

Further analysis of differential metabolites in the liver of Nile tilapia was performed. The level of β-alanine of fish in the G2 and G3 groups were significantly higher than that of the G1 group fish; γ- glutamyl leucine, oleic acid, linoleic acid, and lysoPC levels in the liver of fish in the G3 group were significantly lower than those in the G1 group, while inosinic acid, glucose 6-phosphate, and mannose 6-phosphate levels in fish liver in the G3 group were significantly lower than those in the G1 group. The levels of pyruvic acid in fish liver in the G5 group were significantly higher than those in the G3 group. In addition, the levels of allysine in the liver of fish in the G2, G3, G4, and G5 groups were significantly lower than those in the G6 group, while the levels of adenosine in the liver of fish in the G2, G3, and G5 groups were significantly lower than those in the G6 group.

#### 3.2.2. Metabolic KEEG Function Enrichment

**Figure 7:**
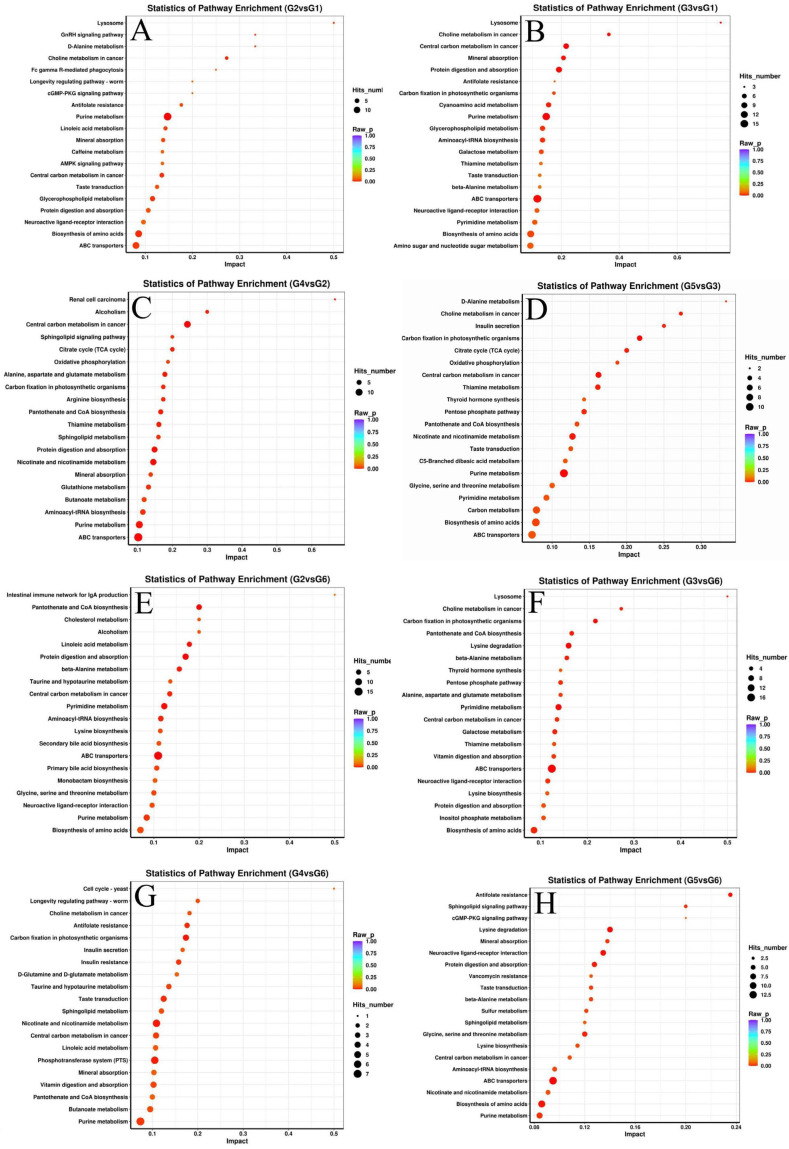
KEGG enrichment of different metabolites in the liver of fish from different comparison groups. (**A**,**B**) KEGG enrichment analysis of hepatic differential metabolites in G2 or G3 and G1 groups; (**C**) KEGG enrichment analysis of differential metabolites in G2 and G4 groups; (**D**) KEGG enrichment analysis of differential metabolites in G3 and G5 groups; (**E**–**H**) KEGG enrichment analysis of differential metabolites in G2, G3, G4 or G5 and G6 groups.

The functional enrichment results of the differential metabolites of Nile tilapia in KEGG are shown in Figure 7. The results showed that 245 differential metabolites in the liver of fish in the G2 and G1 groups were mainly enriched in the lysosomes, GnRH signaling pathways, and D-alanine metabolism. The 323 differential metabolites in the liver of the G3 and G1 groups of fish were mainly enriched in the lysosomes, mineral absorption, and protein digestion and absorption. The 258 differential metabolites in the liver of fish in the G4 and G2 groups were mainly enriched in the sphingolipid signaling pathway and the tricarboxylic acid cycle (TCA cycle). The 231 differential metabolites in the G5 and G3 groups of fish were mainly enriched in the D-Alanine metabolism, insulin secretion, and the citrate cycle (TCA cycle). The 274 differential metabolites in the liver of the G2 and G6 groups of fish were mainly enriched in cholesterol metabolism, pantothenate, and coenzyme A biosynthesis. The 246 differential metabolites in the liver of the G3 and G6 groups of fish were mainly enriched in lysosomes and choline metabolism in cancer. The 200 differential metabolites in the liver of fish from the G4 and G6 groups were mainly enriched in the cell cycle yeast and the longevity regulating pathway worm. The 202 differential metabolites between the G5 and G6 groups of fish were mainly enriched in antifolate resistance, sphingolipids signaling pathway, and cGMP-PKG signaling pathway. The results showed that the experimental crisping formula diets (G2–G5) significantly affected amino acid metabolism, mineral absorption, and protein digestion and absorption, but these metabolic pathways were not significantly different between the experimental crisping formula diets and the commercial crisping formula diet group.

### 3.3. Combined Analysis of Main Differential Metabolites and Functional Genes

**Figure 8:**
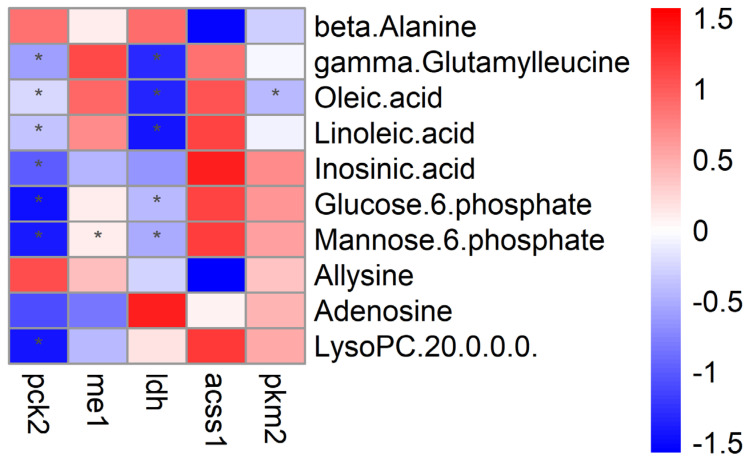
Heatmap of the correlation between the liver major differential metabolites and functional genes in Tilapia. The symbol * indicates significant relevance (*p* < 0.05).

The combined analysis results of the main differential metabolites in the liver and functional genes are shown in Figure 8. There was a significant negative correlation between the liver functional genes *ldh* and *pck2* with γ-glutamine leucine, oleic acid, linoleic acid, glucose 6-phosphate, and mannose 6-phosphate.

## 4. Discussion

Although broad beans can cause muscle fragility, their lack of sulfur-containing amino acids, especially cysteine and methionine, as well as the presence of antinutritional factors, will affect their biological value and limit their use [20,21]. Due to the antinutritional factors in broad beans, they may hinder protein digestion, leading to a decrease in protein bioavailability [21,22]. However, our previous research has shown that there was no adverse effect on the growth performance of Nile tilapia fed with a broad bean crispy diet [19]. We even saw some improvement effects in WGR and SGR and obtained higher FCR. In addition, there was an increase witnessed in the muscle hardness, adhesiveness, and chewiness of all broad bean-based crisping diets compared with the control. Muscle hardness, springiness, adhesiveness, and chewiness are parameters that directly reflect the meat quality, while hardness and chewiness are key indicators that directly reflect the degree of fish fragility [23]. The supplementation of the crisping package in the diet can further improve muscle hardness, adhesiveness, and chewiness. The results of growth performance and flesh quality indicated that broad beans-based crisping diets can achieve the crisping effect on tilapia muscles and promote growth simultaneously.

### 4.1. Transcriptome Analysis

The liver is the main place of glucose metabolism. Glucose metabolism in the liver is related to the energy metabolism of fish and is also closely related to metabolic processes such as ammonia metabolism and ion balance regulation in the body [24]. Through interaction with metabolic processes such as fat metabolism and ammonia metabolism, the fish body jointly maintains the physiological balance of the fish body, thereby affecting the growth and development of the body [25]. Based on the results of multiomics, they were all enriched in the pathways and processes related to energy metabolism, especially glycolysis and gluconeogenesis. In cells, pyruvate is an important intermediate product of the glucose metabolism process and a precursor for the synthesis of glycerol, fatty acids, etc. [26]. Pyruvate metabolism in the body plays an important role in cellular homeostasis. At present, the body mainly regulates the metabolic flux of pyruvate by regulating pyruvate dehydrogenase (PDH), pyruvate carboxylase (PC), etc., and mitochondrial pyruvate transport carrier (MPC). The pyruvate dehydrogenase complex is involved in the body’s glucose metabolism process, catalyzing the pyruvate formation of acetyl-CoA [27]. As the intermediate product of the glucose metabolism process, the change of pyruvate in the results proved the alteration from glycolysis to gluconeogenesis.

The main nutrient of broad beans is starch, and different starch levels in the feed will affect the glucose metabolism of tilapia and significantly affect the growth performance of fish [28,29]. The supplementation of broad beans induced the metabolism reprogramming of tilapia; the glycolysis process was inhibited as gluconeogenesis was promoted. Fish started to synthesize more glucose stored as muscle energy stock (glycogen or other forms). Meanwhile, it also means the transformation of energy sources, such as aerobic glycolysis, towards anaerobic glucose metabolism. This metabolism reprogramming can also be seen in promoting tumor growth and cell proliferation, maintaining tumor cell proliferation in vitro and growth in vivo [30]. Strikingly, except for tumors, this kind of metabolism change is also a common key foundation in muscle cell growth. It was also reported that cardiomyocyte hypertrophic growth related to similar metabolic reprogramming in response to cardiac stress [31] and glucose synthesis increase induced by *pck2* involved in the proliferation of vascular smooth muscle cells in neointimal hyperplasia [32]. It was demonstrated by Brown et al. [33] that the growth hormone (GH) or β-adrenergic agonist greatly enhanced glucose anabolism to promote the growth of porcine skeletal muscle. Meanwhile, it has been found that glycolysis metabolic reprogramming precedes muscle hypertrophy, which demonstrates that metabolic reprogramming could be an initiator of muscle structural remodeling [34], and it also should be the vital foundation of the crisping mechanism.

It should be emphasized that for metabolic reprogramming, in addition to being stimulated by broad bean, other ingredients in the crisping function package were also instrumental. In this study, the crisping function package mainly contained bile acids and taurine, which have the effect of regulating the homeostasis of glucose in aquatic animals. Studies have found that the addition of taurine to high-glucose feed significantly improves the liver glycolysis and glycogen synthesis capacity of turbot and maintains the normal blood glucose level of fish [35]. The addition of taurine to feed can improve hepatic glycogen synthesis and inhibit gluconeogenesis [36]. Bile acids activate the liver bile acid-FXR signaling pathway in largemouth bass, thereby enhancing glycogen synthesis [36,37]. Studies have found that selenium promotes glucose transport and regulates the activities of key enzymes in glucose metabolism, such as glycolysis, gluconeogenesis, and glycogen synthesis [38,39]. Moderate amounts of selenium in feed can maintain homeostasis in glucose metabolism by regulating glycolysis and gluconeogenesis in *Megalobrama amblycephala* [40]. This suggests that functional packs may affect the glucose metabolism of tilapia through taurine, bile acids, and yeast selenium, thereby affecting the growth of fish muscle fiber.

The present study also found a similar phenomenon in immune response as the findings in the research of other crispy fish. Pathways enriched include the Phagosome, NOD-like receptor signaling pathway, toll-like receptor signaling pathway, and Herpes simplex virus 1 infection. Strong oxidative stress status and immune response are important phenomena occurring in fish fed with broad beans. In both grass carp and Yellow River carp research, they have found that broad beans can cause oxidative stress in fish and further regulate muscle texture through ROS mediation, which is an important basis for the crisping mechanism [9,41]. Long-term exposure to high levels of ROS causes changes in the fiber structure and function of muscles, specifically manifested in the down-regulation of myosin and actin, which means actin-myosin interaction was impaired and finally led to a decrease in muscle fiber diameter [42,43]. In addition, the generation of high ROS also resulted in mitochondrial damage [7]. The high-level activation of the Phagosome pathway in the present study is consistent with the results in other studies, where autophagy-related mitochondrial damage is clear after being fed a broad beans-based diet.

Through the transcriptome analysis of crispy grass carp, Xu et al. [41] found many significant differentially expressed genes related to immune functions, including lysophosphatidic acid acyltransferase activity, lymphangiogenesis, MHC class II protein complex, regulation of cytokine production, interleukin-2 production, among others. Subsequently, a further study of genome-wide methylation analysis by Li et al. [44] also obtained corresponding results; many immune-related genes were found to have differential methylation in crispy grass carp, including nfkb1, TNF-α, MHC (class I and II), INF-γ, some cytokine synthesis inhibitory factors (IL-4, IL-10, IL-12, etc.).

NOD-like receptor signaling pathway and Toll-like receptor signaling pathway all belong to pattern-recognition receptors (PRRs). In transmembrane and cytoplasm, these two receptors combine to play a crucial role in the innate immune response by recognizing damage-associated molecular patterns (DAMPs) and pathogen-associated molecular patterns (PAMPs) [45]. Activated PRRs can induce inflammasome formation, signaling transduction, transcription activation, and autophagy [46]. Notably, in addition to being activated by pathogens, PRRs can also be triggered by self-derived DAMPs like glucose [47]. In our study, these two pathways are probably caused by the high glucose content of fish. Furthermore, the NOD-like receptor signaling pathway and the Toll-like receptor signaling pathway both can also further activate downstream pathways, including the NF-kB pathway and MAPK signaling pathway, which leads to proinflammatory cytokine secretion and then cause widespread inflammatory response [48,49]. The MAPK signaling pathway was enriched in the present study, especially in the G4 and G5 diet groups, which added a crisping package; it indicated that the immune response resulting in muscle structure change was mainly brought out by the MAPK signaling pathway. In addition to the MAPK signaling pathway, the ErbB signaling pathway was found in crisping function package groups. Signaling mediated by ErbB has been proven to regulate the formation of neuromuscular synapses and promote the development of muscle spindles and myoblasts [50,51]. Also, hyperglycemia causes an increase in skeletal muscle fibrosis, and this increase is associated with the activation of the ErbB [52]. Based on that, it can be speculated that the crisping mechanism of Tilapia will have a similar regulating process. Meanwhile, the ErbB signaling pathway is also the upstream pathway of the MAPK signaling pathway [53]. To summarize, the additive of the crisping function package got a better crisping performance by further activating the ErbB signaling pathway and MAPK signaling pathway.

Broad bean diets can also activate a virus immune pathway (Herpes simplex virus 1 infection pathway). Herpes simplex virus 1 infection can cause the activation of the innate immune response by the interaction between cellular PRR and viral PAMP [54], but there was no virus infection, so the Herpes simplex virus 1 infection pathway probably also activated the innate immune response through the Glucose DAMP- cellular PRR interaction mentioned above.

### 4.2. Metabolomic Analysis

In this study, after Nile tilapia ingested a compound diet containing broad beans, the metabolites in the liver underwent obvious changes, and most of the differential metabolites were related to amino acids, glucose, and lipids. Previous studies also reported that broad beans can affect the metabolism of fish, especially glucose and lipid metabolism [55]. Further analysis of differential metabolites found that compared with the basic diet (G1), both broad bean-based compound diets (G2 and G3) significantly increased liver β-alanine levels, reduced liver γ-glutamyl leucine and hemolytic lecithin levels. γ-glutamyl leucine, a product of the incomplete breakdown or digestion of proteins, consists of γ-glutamic acid and leucine [56]. High levels of γ-glutamyl leucine are associated with oxidative stress [57]. γ-glutamyl amino acids are an important part of the γ-glutamyl cycle, and the γ-glutamyl cycle is mainly responsibility for the transmembrane transport of amino acids [58]. According to that, decreasing the level of γ-glutamyl leucine means more Leucine is being transported across the membrane and utilized. Leucine has proved to be an excellent supplement for improving the muscle texture of fish. High leucine supplementation got a higher muscle texture performance (hardness, springiness, resilience, and chewiness), small-sized fiber ratio, fiber density, and sarcomere lengths in fish, and this promotion may be related to the activation of BCKDH and AMPK [59]. Deng et al. [60] also reported that optimum leucine improved muscle quality by enhancing the antioxidant capacity.

Studies have shown that the lysolecithin level is strongly associated with oxidative stress and inflammation [61]. Hemolytic lecithin has also been reported as a mediator of FFA-induced apoptosis in hepatocyte lipocytes [62]. β-Alanine is an important non-essential amino acid that exerts antioxidant, anti-free radical, anti-aging, and immunity-enhancing effects in the body by combining with histidine to produce carnosine [63,64]. Elevated levels of β-alanine indicate that the broad bean diet also promoted the synthesis or deposition of antioxidant-related metabolites in Nile tilapia liver. Compared with the commercial crisping compound diet (G6), the contents of liver aldehyde, lysine, and adenosine were reduced in the broad bean feed group with or without a functional package. Under normal conditions, adenosine content is low, but when the body or tissue is damaged in a state of inflammation and hypoxia, the adenosine content increases rapidly, thereby suppressing the immune response and preventing excessive damage to the body or tissue [65]. Aldehyde lysine is a specific marker of protein oxidation, and the high amount of ROS and severe oxidative stress can lead to increased levels [66].

In this study, a high broad bean-level compound diet (G3) also significantly reduced liver glucose-6-phosphate and mannose-6-phosphate levels compared with a broad bean-free basic compound diet. Glucose-6-phosphate is an extremely important intermediate product of pentose phosphate and glycolysis pathway, in which glucose-6-phosphate is produced by hexokinase catalyzed glucose and then catalyzed by phosphoglucose isomerase to form glucose-6-phosphate, and then further decomposed. The pentose phosphate pathway is initiated by glucose-6-phosphate, which is also the main pathway to produce NADPH. In addition, glucose-6-phosphate can also be converted into starch or glycogen to be stored [67]. Mannose-6-phosphate can inhibit hexokinase, phosphoglucose isomerase, and glucose-6-phosphate dehydrogenase, three enzymes involved in glucose metabolism, thereby impairing the further metabolism of glucose in glycolysis, as well as the pentose phosphate pathway, the tricarboxylic acid cycle, and glycan synthesis [68]. In this study, liver glucose-6-phosphate and mannose-6-phosphate levels decreased after the tilapia ingestion of broad beans, suggesting that broad beans may affect pentose phosphate and glycolytic pathways. This aspect may be related to the broad bean’s antinutritional factors. For example, the vicioside in broad beans, which is a competitive inhibitor of glucose-6-phosphate, may affect the body’s glucose metabolism [69]. On the other hand, it may be related to the higher starch content of broad beans.

In addition, broad bean diets (G2 and G3) in this study also reduced liver oleic acid and linoleic acid levels. In grass carp, it was found that the linoleic acid content of muscle was also significantly reduced after 8 weeks of feeding broad beans [70]. He et al. [71] added DHA to the diet and found it enhanced the textural firmness of Common Carp. It has been proved that low saturated fatty acids and high unsaturated fatty acid proportions result in high muscle fiber size, and the glycolytic fiber type is related to the ratio of n-6/n-3 fatty acids [72]. In brief, fatty acid composition significantly affects muscle texture. In another research about muscle lipids, they found that muscle fiber sizes have a negative relationship with C18:0 (oleic acid and linoleic acid included) [73]. That explained how the mechanism of the decrease of liver oleic acid and linoleic acid levels leads to the muscle fiber size change.

It was found in this study that the broad bean formula diet not only affected the glucose and lipid metabolism pathway but also mineral absorption and protein digestion and absorption, especially under high proportions of broad bean used. Research has reported that broad beans contain various antinutritional factors such as tannins, phytic acid, protease inhibitors, broad bean agglutinin, and nestin glycosides. When digested, these antinutritional factors may bind to certain proteins in the diet, reducing the digestibility of proteins. Moreover, these antinutritional factors may also bind to digestive enzymes (such as trypsin) to form complexes, reducing the activity of digestive enzymes and further affecting the digestion and absorption of nutrients [74]. Tannins can have strong interactions with proteins, resulting in the formation of difficult-to-digest polymer precipitates and reducing the animal utilization of proteins [75]. The protease inhibitors in broad beans form inactive ligands with chymotrypsin or trypsin, leading to a decrease in the digestibility of proteins in the intestine [76]. In addition, broad beans contain a high content of phytic acid, which combines with positively charged metal ions such as calcium, iron, and zinc to form insoluble phytates, seriously affecting the absorption of mineral elements by animals. The complexation of these metal ions further inhibits enzyme activity, thereby affecting the absorption of egg-white matter [77]. In this study, it was also found that the crisping functional package significantly affected the TCA cycle, indicating that the crisping functional package may improve body metabolism by regulating TCA cycle metabolism. It was also found in mice that adding taurine can improve the utilization of glucose by myoblasts and improve TCA cycling metabolism [78]. In summary, high broad bean feed has a significant impact on the mineral absorption, protein digestion, and absorption-related metabolic pathways of tilapia. The crisping function package may improve the body’s metabolism by regulating TCA cycle metabolism.

In summary, broad bean supplementation mainly results in the metabolism reprogramming characterized by the inhibition of glucose metabolism, influencing the metabolism of glucose, lipids, minerals, and protein of fish and ultimately achieving changes in muscle structure.

### 4.3. Correlation Analysis Between Main Metabolites and Liver Function Genes

The results of multiomics both showed that a broad bean compound diet affected the glucose metabolism of tilapia liver. Combined with metabolomics and transcriptomics association analysis, it was characterized that the levels of *pck2* and *ldh* gene were the essential factors to affect glucose metabolism by controlling the content of glucose-6-phosphate. Gluconeogenesis is a glucose metabolism pathway that uses non-carbohydrate precursors (mainly glutamine, alanine, glycerol, etc.) to synthesize glucose. Phosphoenolpyruvate carboxykinase (PCK) is a key enzyme in the gluconeogenesis pathway, with two isoforms in the body: *pck1* and *pck2* [79]. Among them, *pck2* plays an extremely important regulatory role in gluconeogenesis, and its activation can also influence the TCA cycle flux and inhibit glycolysis [34,80]. In addition, in fish, LDH can catalyze the production of lactic acid by pyruvate under anaerobic conditions, realizing the conversion of lactic acid and pyruvate [81]. We can conclude that a broad bean-based crisping compound diet induces the glucose metabolic reprogram through *pck2* and *ldh* genes upregulating, generating sufficient glucose to meet the energy requirement caused by muscle hyperplasia. The metabolic reprogram was achieved by affecting the gluconeogenesis pathway of tilapia liver by upregulating *pck2* expression and *ldh* gene upregulating to mediate anaerobic glycolysis for alternative energy supply at the same time. In the meantime, combined with the results of the transcriptome above, increasing glucose driven by *pck2* and *ldh* genes can also act as a DAMP to be recognized by cellular PRRs, activating the innate immune response and finally resulting in muscle change.

In addition, *pck2* and *ldh* are also significantly negatively correlated with the changes in liver γ-glutamyl leucine, oleic acid, and linoleic acid. High γ-glutamyl leucine has proved to be related to low blood glucose concentration [82]. Hence, the *pck2* and *ldh* genes probably decrease the level of γ-glutamyl leucine through glucose metabolism regulation. In other words, it indicated that *pck2* and *ldh* genes promote the γ-amma-glutamyl cycle after fed a broad bean-based diet. That benefited the transportation of amino acids and their utilization, especially leucine. Finally, the flesh quality of fish improved by the absorbed leucine. Besides, the strong link between the decreased content of oleic acid and linoleic acid and the high expression of *pck2* and *ldh* genes implied the promote β-oxidation of fatty acids. Fatty acid composition change was also seen in the research on the crisping mechanism of other fishes. For example, a significantly higher n-6 PUFA content and the ratio of DHA/EPA in muscle were seen in Yellow River carp fed with broad bean.

## 5. Conclusions

In brief, the designed crisping diets for Nile tilapia can effectively make tilapia muscle crispy. The ingestion of broad bean-based diets induced metabolic reprogramming dominated by glycolytic metabolism inhibition in fish, and metabolic reprogramming is the initiator of muscle structural remodeling through activating the further immune response and oxidative stress. In addition, the addition of the crisping package further activated the ErbB signaling pathway and downstream MAPK signaling pathway, promoting muscle fiber development and growth.

Broad bean-based diets produced a marked effect by inducing metabolism reprogramming, including glucose, fatty acid, and amino acid metabolism; *pck2* and *ldh* played main roles in the crisping molecular mechanism. They achieved crisping effects through promoting gluconeogenesis and anaerobic glycolysis to generate sufficient glucose to meet the energy requirement caused by muscle hyperplasia, Meanwhile, glucose as a DAMP is recognized by cellular PRRs activating the innate immune response. At the same time, the genes *pck2* and *ldh* also led to an increase in the transportation and utilization of leucine, changing the composition of fatty acids and finally resulting in muscle change.

## Data Availability

The data presented in this study are available on request from the corresponding author.
